# 6-Shogaol-Rich Extract from Ginger Up-Regulates the Antioxidant Defense Systems in Cells and Mice 

**DOI:** 10.3390/molecules17078037

**Published:** 2012-07-04

**Authors:** Min-Ji Bak, Seon Ok, Mira Jun, Woo-Sik Jeong

**Affiliations:** 1 Department of Food & Life Sciences, College of Biomedical Science & Engineering, Inje University, Gimhae 621-749, Korea; Email: redapplemj@hanmail.net (M.-J.B.); littleok02@nate.com (S.O.); 2 Department of Pharmacy, Kyungsung University, Busan 808-736, Korea; 3 Department of Food Science & Nutrition, Dong-A University, Busan 604-714, Korea; Email: mjun@dau.ac.kr

**Keywords:** ginger, 6-shogaol, Nrf2, antioxidant response element (ARE), antioxidant defense, MAPK, chemoprevention, cytoprotection, hemeoxygenase 1, antioxidant enzyme

## Abstract

The rhizome of ginger (*Zingiber officinale* Roscoe) is known to have several bioactive compounds including gingerols and shogaols which possess beneficial health properties such as anti-inflammatory and chemopreventive effects. Based on recent observations that 6-shogaol may have more potent bioactivity than 6-gingerol, we obtained a 6-shogaol-rich extract from ginger and examined its effects on the nuclear factor E2-related factor2 (Nrf2)/antioxidant response element (ARE) pathway *in vitro* and *in vivo*. 6-Shogaol-rich extract was produced by extracting ginger powder with 95% ethanol at 80 °C after drying at 80 °C (GEE8080). GEE8080 contained over 6-fold more 6-shogaol compared to the room temperature extract (GEE80RT). In HepG2 cells, GEE8080 displayed much stronger inductions of ARE-reporter gene activity and Nrf2 expression than GEE80RT. GEE8080 stimulated phosphorylations of mitogen-activated protein kinases (MAPKs) such as ERK, JNK, and p38. Moreover, the GEE8080-induced expressions of Nrf2 and HO-1 were attenuated by treatments of SB202190 (a p38 specific inhibitor) and LY294002 (an Akt specific inhibitor). In a mouse model, the GEE8080 decreased the diethylnitrosamine (DEN)-mediated elevations of serum aspartate transaminase and alanine transaminase as well as the DEN-induced hepatic lipid peroxidation. Inductions of Nrf2 and HO-1 by GEE8080 were also confirmed in the mice. In addition, the administration of GEE8080 to the mice also restored the DEN-reduced activity and protein expression of hepatic antioxidant enzymes such as superoxide dismutase, glutathione peroxidase and catalase. In conclusion, GEE8080, a 6-shogaol-rich ginger extract, may enhance antioxidant defense mechanism through the induction of Nrf2 and HO-1 regulated by p38 MAPK and PI3k/Akt pathway *in vitro* and *in vivo*.

## 1. Introduction

The cellular defense mechanism through induction of phase II detoxifying and/or antioxidant stress responsive genes is extremely important in protection of cells/tissues against both toxic chemicals and the resulting oxidative stress [1,2]. Recent findings support the key roles of the antioxidant response element (ARE) in regulating the expressions of some phase II and antioxidant enzymes such as heme oxygenase-1 (HO-1), glutathione S-transferase (GST), and NAD(P)H: quinine oxidoreductase 1 (NQO1) by phenolic antioxidants and other naturally occurring cancer chemopreventive agents [1,3]. These enzymes protect cells and/or tissues from carcinogens that can initiate carcinogenic process [1,4,5]. Upon exposure of cells to inducers such as oxidative stress and chemopreventive agents, nuclear factor E2-related factor 2 (Nrf2) translocated from the cytosol to the nucleus and binds to ARE in the promoter region of the phase II and antioxidant genes [4]. Mitogen-activated protein kinases (MAPK) such as extracellular signal-regulated kinase (ERK), c-Jun N-terminal kinase (JNK) and p38 have been shown to regulate Nrf2-ARE mediated gene expressions [2,6]. 

The rhizome of ginger (*Zingiber officinale* Roscoe) originated in Southeast Asia and is nowadays widely spread around the World. It has been reported as a traditional medicinal herb for thousands of year to treat many diseases such as gastrointestinal, stomachic, and rheumatic disorders [7]. The anti-inflammatory and antithrombotic effects of ginger have been associated with its ability to inhibit prostaglandin biosynthesis [8]. Several bioactive compounds have been identified in ginger, including gingerols, shogaols, paradols, and zingerones [7,9,10]. Of the ginger compounds, 6-gingerol, the most abundant bioactive compound in ginger, has been extensively studied for its various pharmacological effects including anti-inflammatory, analgesic, antipyretic, chemopreventive, and antioxidant properties [11,12,13,14,15]. Interestingly, recent studies have demonstrated that 6-shogaol, a minor component of ginger, might be more biologically active than 6-gingerol [16,17,18,19]. Bhattarai *et al.* [20] have recently reported that 6-gingerol can be degraded to 6-shogaol in a model system such as acidic conditions and high temperature. In the present study, we applied this observation to a real extraction process, produced a 6-shogaol-rich extract, and evaluated its regulatory roles in cellular defense mechanisms, in particular its ability to induce Nrf2/ARE-mediated gene expressions in cells and an animal model. 

## 2. Results and Discussion

### 2.1. 6-Shogaol Content of the Ginger Extract

**Figure 1:**
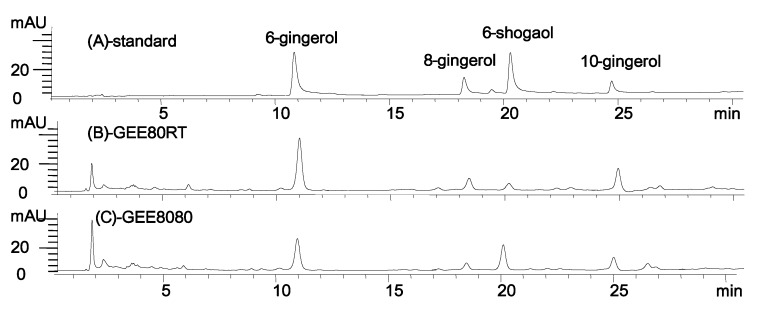
HPLC chromatograms of standard ginger compounds and representative ginger extracts. (**A**) standard compounds of ginger; (**B**) GEE80RT, ginger extract extracted with 95% at room temperature after drying at 80 °C; (**C**) GEE8080, ginger extract extracted with 95% ethanol at 80 °C after drying at 80 °C.

The extraction of dried ginger with 95% ethanol at 80 °C (GEE8080, ginger extract extracted with 95% ethanol at 80 °C after drying at 80 °C) resulted in a sharp increase in 6-shogaol content (Figure 1). During this extraction, the 6-shogaol content was elevated to about 6-fold (21 mg/g dry matter) (GEE8080) compared to the same extraction at room temperature (3.5 mg/g dry matter, GEE80RT, ginger extract extracted with 95% at room temperature after drying at 80 °C). This change in 6-shogaol content might be a result of dehydration of 6-gingerol to 6-shogaol at high temperature during extraction [20]. The detailed optimization process and the results on the extraction will be reported separately. 

### 2.2. Cell Viability in HepG2 Cells

**Figure 2:**
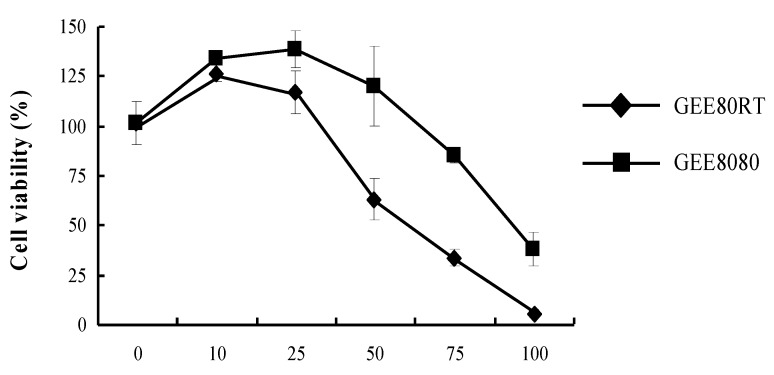
Effects of ginger extracts on cell viability of HepG2 cells. HepG2 cells were maintained in 24-well plates at a density of 10^5^ cells/well. Cells were treated with different doses for 24 h. The percentage of viable cells was calculated as a ratio of 570 nm of treated cells versus control cells (treated with 0.1% DMSO vehicle).

The effects of GEE80RT and GEE8080 on the viability of HepG2 cells were determined by a 3-(4,5-dimethylthiazol-2-yl)-2,5-diphenyltetrazolium bromide (MTT) assay after 24 h treatment. The data were expressed as percent cell viability compared to that of control (Figure 2). The concentrations of the treatments GEE80 and GEE8080 varied from 10 to 100 µg/mL. Treatments with both ginger extracts of GEE80RT and GEE8080 resulted in a dose-dependent inhibition on HepG2 cell viability and GEE80RT displayed more potent inhibitory activity against cell viability than GEE8080. GEE8080 did not show the cell viability inhibition at the concentrations up to 50 µg/mL while GEE80RT at 50 µg/mL inhibited the cell viability by about 40%. Based on the cell viability data, further biological assays were performed at concentrations below 50 µg/mL. 

### 2.3. ARE-Luciferase Reporter Gene Activity in HepG2 Cells

**Figure 3:**
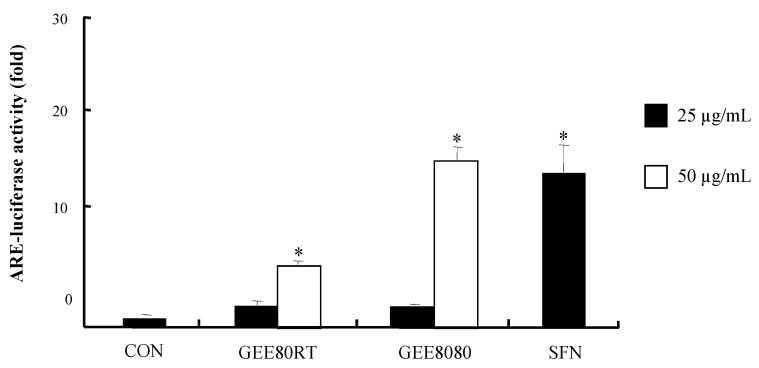
ARE-luciferase activity by ginger extracts in HepG2-C8 cells. HepG2-C8 cells, stably transfected with pARE-T1-luciferase reporter gene, were treated with vehicle (DMSO, 0.1%) or each ginger extract for 12 h. Luciferase activity was normalized with protein content and expressed as fold-induction against vehicle-treated control. Sulforaphane (SFN) was used as a positive control. Values are represented as mean ± SD (n = 5), * significantly different from vehicle treatment, respectively, *p *< 0.05.

The ARE-reporter gene assay can be a useful screening tool to examine unknown cytoprotective agents due to the critical region of ARE sequence in phase II and antioxidant enzymes involved in cellular defense mechanisms [1]. To investigate the effects of ginger extracts (both GEE80RT and GEE8080) on ARE transcriptional activity, we treated the extracts in HepG2-ARE-C8 cells, stably transfected with the pARE-T1-Luciferase reporter gene using HepG2 cells. As shown in Figure 3, the treatments with ginger extracts showed strong inductions of ARE-luciferase activity. The induction of ARE-luciferase activity by GEE8080 at 50 µg/mL was much more potent than GEE80RT and the induction was similar to that of the positive control sulforapane (SFN). SFN has received an intense attention for its cancer-chemopreventive potential because it is one of the most potent inducers of phase II detoxifying enzymes among many natural compounds [21]. 

### 2.4. Induction of Nrf2 and HO-1 Expression in HepG2 Cells

**Figure 4:**
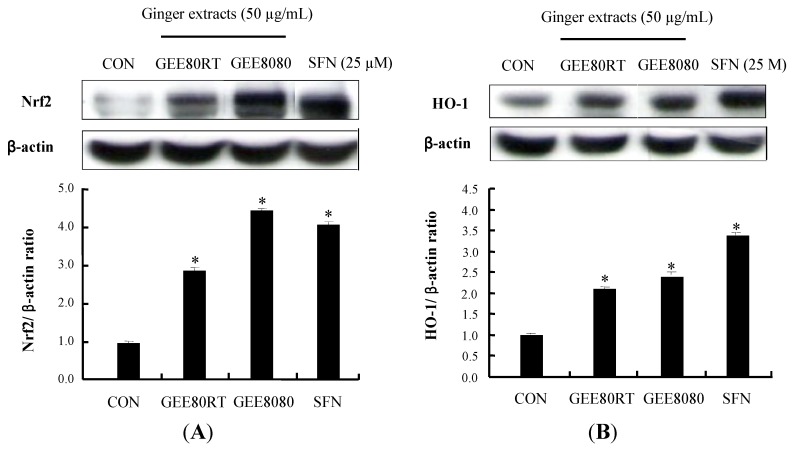
Effects of ginger extracts on Nrf2 and HO-1 protein expression. (**A**) Nrf2 expression levels after treatments with ginger extracts. HepG2 cells were treated with 50 µg/mL for 2 h; (**B**) HO-1 expression levels after treatments with ginger extracts. HepG2 cells were treated with 50 µg/mL ginger extracts for 24 h. Induction fold of Nrf2 and HO-1 protein was calculated as the intensity of the treated samples relative to that of the control by densitometry. Sulforaphane (SFN) was used as a positive control. The blots shown are representative of three independent experiments with similar results. Values are means ± SD; *n *= 3, * *p* < 0.05 *vs*. control.

Nrf2 binds to ARE in the promoter region of phase II detoxifying and antioxidant genes such as HO-1, NQO1 and GST, and stimulates the protein expressions of these genes [22]. HO-1 is believed to play a critical role in the regulation of heme metabolism and in cellular defense system against oxidative stress in cells [23,24]. To determine whether the above observed activation of ARE-reporter gene is associated with expressions of Nrf2 and HO-1, we treated HepG2 cells with GEE80RT and GEE8080. As shown in Figure 4A, both ginger extracts strongly induced the protein expressions of Nrf2 while GEE8080 was the most potent inducer of Nrf2 expression and the induction by GEE8080 (50 µg/mL) was stronger than that by SFN (25 μM). In addition, the GEE8080 treatment resulted in stronger upregulation of the antioxidant enzyme HO-1 expression than GEE80RT treatment (Figure 4B). These results suggest that HO-1 protein expressions by ginger extracts may be mediated by the activation of Nrf2/ARE pathway and the stronger activity of GEE8080 compared to GEE80RT could be related at least in part by the increased amount of 6-shogaol in GEE8080. Many natural extracts such as the extracts from *Ginkgo biloba* [25], broccoli [26] and garlic [27] have been reported to induce the Nrf2/ARE-mediated gene expressions including HO-1. Moreover, several purified phytochemicals including sulforaphane, curcumin, diallyl sulfide and procyanidins have been shown to have similar activities on the pathways in various model systems [28,29,30,31,32].

### 2.5. Phosphorylation of MAPKs and PI3K/Akt Pathway in HepG2 Cells

**Figure 5:**
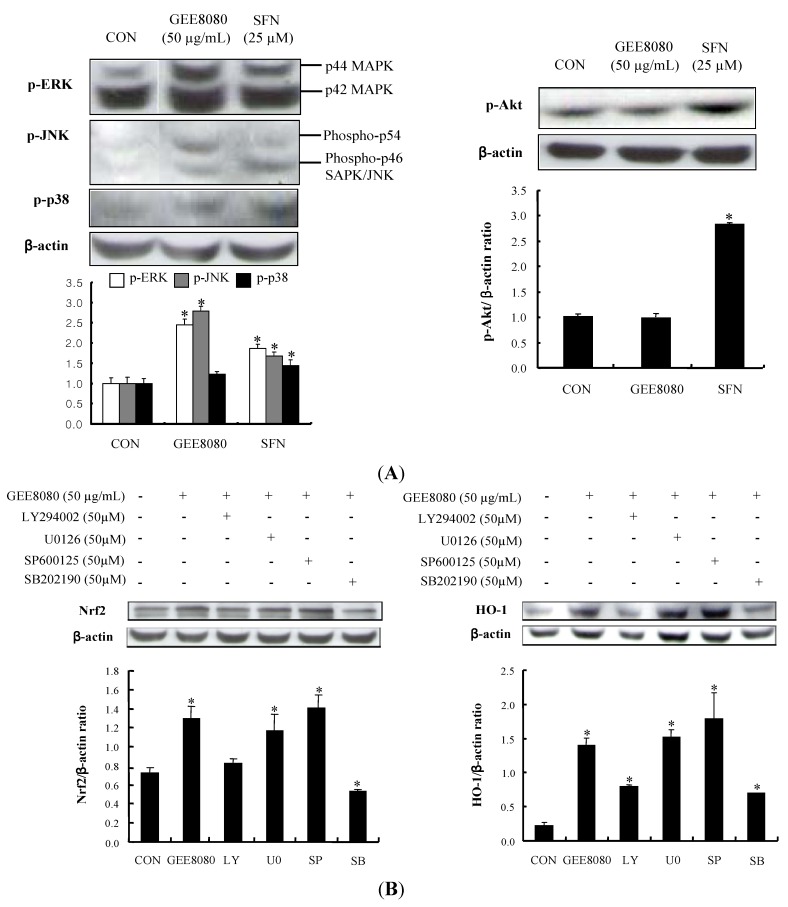
Effects of ginger extracts on the phosphorylations of upstream targets in HepG2 cells. (**A**) Effect of ginger extracts on phosphorylation of MAPKs and Akt. HepG2 cells were treated with 50 µg/mL ginger extracts for 1 h; (**B**) Influence of MAPK inhibitors on GEE8080-induced Nrf2 and HO-1 expression. HepG2 cells pretreated with vehicle or 50 μM U0126 (MEK1/2 inhibitor), SP600125 (JNK inhibitor), SB202190 (p38 inhibitor), and LY294002 (Akt inhibitor) for 1 h prior to treatment with 50 μg/mL GEE8080 for 1 h. The blots are shown representative of three independent experiments with similar results and data represent the means ± S.D. Locations for each concentration marked letters are significantly different at * *p *< 0.05* vs.* control.

MAPKs including ERK, JNK and p38 have been associated with the induction of phase II detoxifying and/or antioxidant enzymes either by oxidative stress stimuli or by chemopreventive agents [2,28,33]. To determine whether a similar signal mechanism is responsible for the up-regulation of Nrf2 and HO-1 expression by ginger extracts in HepG2 cells, we treated GEE8080 in HepG2 cells and examined the phosphorylation levels of three MAPK subfamilies. Treatment with the GEE8080 resulted in the elevated phosphorylations of all three MAPKs (1.57-, 3- and 1.23-fold for ERK, JNK and p38, respectively) while the extract did not affect the Akt phosphorylation (Figure 5A). Chemopreventive procyanidins from wild grape seeds have been shown to induce the phosphorylations of MAPKs in HepG2 cells [32]. Several synthetic chemopreventive compounds such as butylated hydroxyanisole and ethoxyquin also stimulated the phosphorylations of both ERK and JNK, mediated by Nrf2 transactivation activity in HepG2 cells [34] and phase II detoxifying enzymes induction [35]. 

The role of these upstream kinases by GEE8080 was further studied by application with their specific inhibitors; U0126 (a specific inhibitor of MEK1/2), SB202190 (a specific inhibitor of p38), SP600125 (a specific inhibitor of JNK) and LY294002 (a specific inhibitor of PI3K). The results of this experiment showed that the GEE8080-mediated up-regulation of Nrf2 and HO-1 expression was completely blocked by pretreatment with SB202190 and LY294002, whereas the same concentration of SP600125 and U0126 had no significant effect (Figure 5B). These results indicate that GEE8080 induced up-regulation of Nrf2 and HO-1 expression may be through the activation of p38 and PI3K/Akt pathway in HepG2 cells. Several studies have revealed the association of PI3K/Akt pathway in Nrf2 expression: the anthocyanin faction from purple sweet potato induced Nrf2 and Akt activation [36], the phytoestrogen puerarin from the root of *Pueraria lobata* augments cellular antioxidant defense capacity through HO-1 induction via the PI3K/Akt-Nrf2 signaling pathway [37]. 

### 2.6. Serum AST and ALT Activity in DEN-Treated Mice

**Figure 6:**
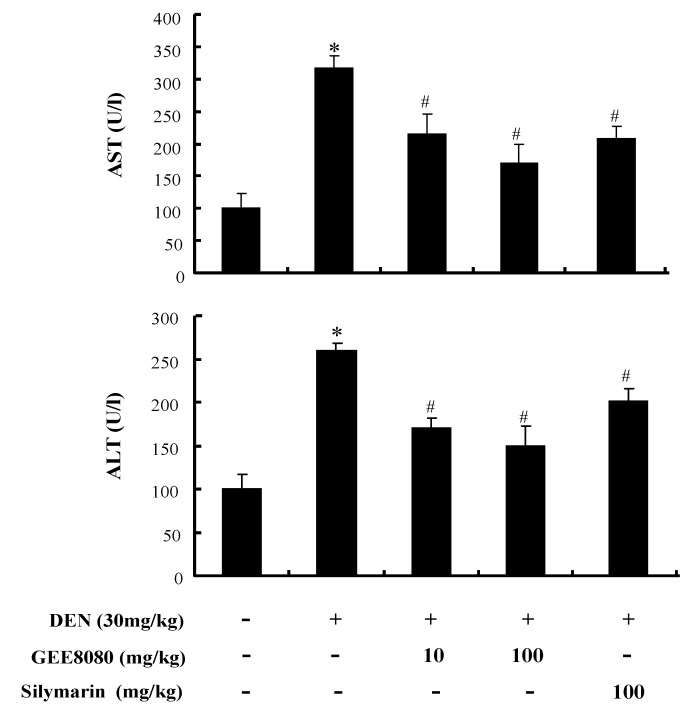
Effects of ginger extracts administration on the activities of serum aspartate aminotransferase and alanine aminotransferase in DEN-treated mice. Each value represents the mean ± S.D for 5 mice. * *p* < 0.05, significantly different from the unstimulated control group. ^#^ *p* < 0.05, significant differences from the DEN-treated group.

The antioxidant defense effect of 6-shogaol-rich ginger extracts was further examined in an animal model. Male Balb/c mice were treated with GEE8080 (10 and 100 mg/kg b.w.) or silymarin (100 mg/kg b.w.), a known hepatoprotective phytochemical as a positive control, and challenged with diethyl-nitrosoamine (DEN, 30 mg/kg b.w.) 3 days per week for 3 weeks. DEN toxicity is primarily associated to an excessive production of free radicals in the liver [38] and is accepted as a model to study the relations among liver necrosis, cancer initiation and replication [39,40]. Serum aspartate aminotransferase (AST) and alanine aminotransferase (ALT) are biomarkers in the diagnosis of hepatic damage because they are released into the circulation after cellular damage [41]. The injection of DEN (30 mg/kg) resulted in dramatic increases of AST and ALT activity in serum compared to control (Figure 6), possibly by DEN-mediated hepatic structural damage [42]. Administration of GEE8080 or silymarin significantly restored serum AST and ALT level in DEN-treated mice. The decrease of AST and ALT was more effective at the higher concentration (100 mg/kg) of GEE8080 than at the lower GEE8080 (10 mg/kg) as well as at the same dose of silymarin. Our results indicate that GEE8080 could be a potential protective agent against acute hepatic damage by oxidative stress.

### 2.7. Hepatic TBARS Content in DEN-Treated Mice

**Figure 7:**
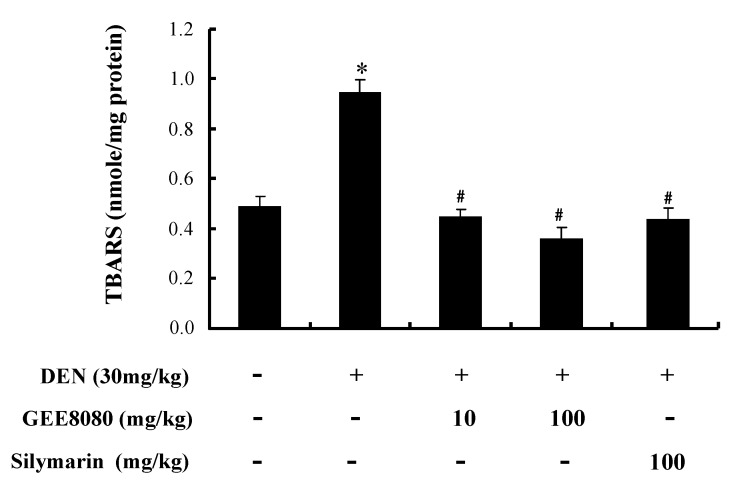
Effects of ginger extracts administration on the activities of hepatic TBARS content in DEN-treated mice. Each value represents the mean ± S.D for 5 mice. * *p* < 0.05, significantly different from the unstimulated control group. ^#^ *p* < 0.05, significant differences from the DEN-treated group.

The extent of lipid peroxidation in hepatic tissues was determined by measuring thiobarbituric acid reactive substances (TBARS) in DEN-treated mice. Since cell membranes consist primarily of lipids, uncontrolled lipid peroxidation may cause cell injury or death via DNA damage and directly inhibiting proteins [43]. Experimental studies have also suggested an inverse relationship between lipid peroxidation and liver injury [44,45]. As shown as Figure 7, DEN treatment increased hepatic TBARS levels about 2-fold compared to vehicle-treated control. Administration of GEE8080 and silymarin to the DEN-treated mice completely inhibited the DEN-induced hepatic lipid peroxidation. Similar to the results from AST and ALT experiment, GEE8080 at 100 mg/kg was more effective in inhibiting lipid peroxidation than the lower dose (10 mg/kg) and silymarin. Several phytochemicals have been shown to inhibit lipid peroxidation as indicated by TBARS value, suggesting their ability to protect liver tissues from free radical damage [1,27,46]. 

### 2.8. Nrf2 and HO-1 Expression in the Liver of DEN-Induced Mice

**Figure 8:**
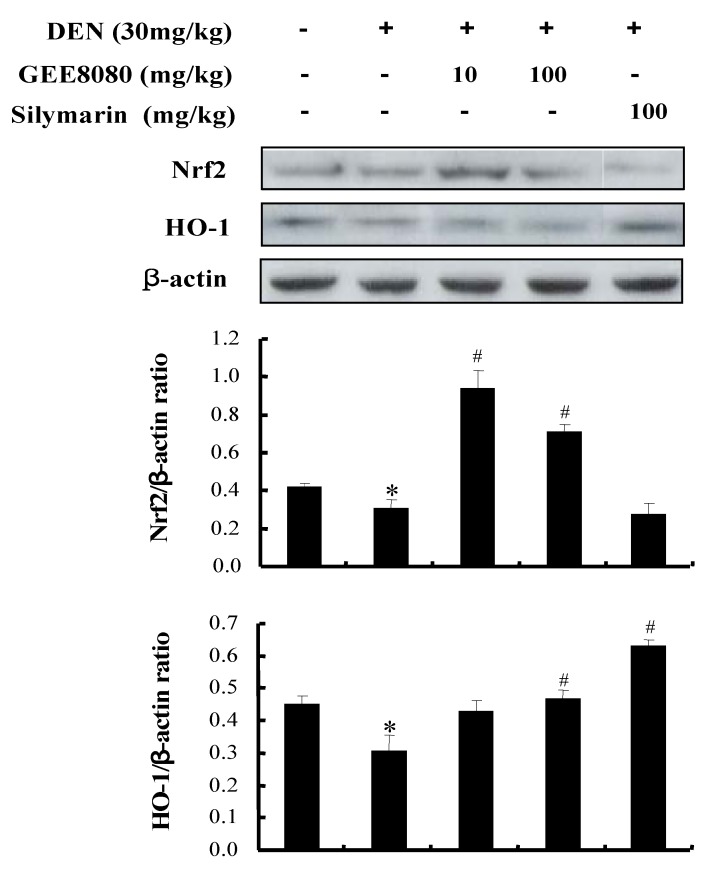
Effects of ginger extracts on Nrf2 and HO-1 protein expression in DEN-induced mice. Each value represents the mean ± S. D. for 4 mice. * *p* < 0.05, significantly different from the unstimulated control group. # *p* < 0.05, significant differences from the DEN-treated group.

Nrf2 is a transcription factor that regulates the expression of antioxidant enzymes in response to oxidative stress [47]. HO-1 mediated cytoprotection has been shown to be critical for tissues that are vulnerable to oxidative stress [48]. Therefore, HO-1 is recognized as an important target of a number of chemopreventive and cytoprotective agents [49]. The activation of Nrf2 and HO-1 by GEE8080 observed at the cell line model was further examined in animal model system. As shown in Figure 8, the treatment DEN significantly attenuated the protein expression of Nrf2 and antioxidant enzyme HO-1. Administration of GEE8080 to the mice dramatically increased the Nrf2 expression. In addition, the DEN-attenuated HO-1 expression was restored by GEE8080. These data suggest a possible involvement of Nrf2 and Nrf2-mediated HO-1 expression by GEE8080 which may contribute to hepatic protection against DEN toxicity in mice. 6-Gingerol, a major constituent in ginger, has been shown to have hepatoprotective effects in acetaminophen-induced hepatotoxic mice through lowering hepatic markers such as AST and ALT [50]. Our study reports for the first time the hepatoprotective activity of 6-shogaol-rich ginger extract via Nrf2/ARE-mediated antioxidant defense mechanism.

### 2.9. Antioxidant Enzymes Activity and Expression in the Liver of DEN-Induced Mice

**Figure 9:**
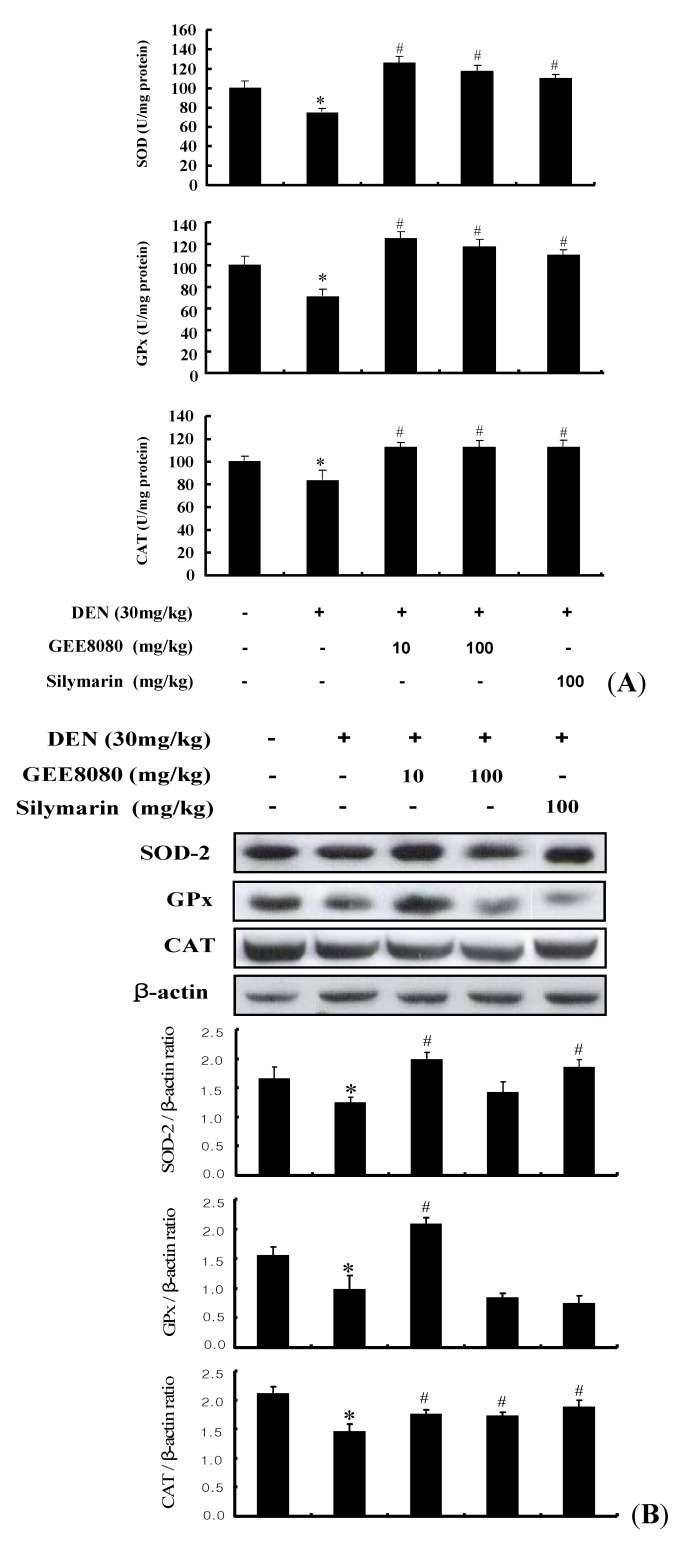
Effects of ginger extracts on (**A**) hepatic SOD, GPx and CAT activities and (**B**) protein expressions in DEN-induced mice. Each value represents the mean ± S.D. for 4 mice. * *p* < 0.05, significantly different from the unstimulated control group. ^#^ *p* < 0.05, significant differences from the DEN-treated group.

In order to investigate whether the Nrf2-inducing activity of GEE8080 is related to its ability to induce the endogenous antioxidant enzymes, we examined its effects on the activity and expression of antioxidant enzymes such as superoxide dismutase (SOD), glutathione peroxidase (GPx) and catalase (CAT) in the mice treated with DEN. The antioxidant enzymes such as SOD, GPx and CAT protect membrane and cytosolic components against damage caused by free radicals during carcinogenesis [51]. Decreased lipid peroxidation associated with an enhanced glutathione (GSH) and GSH-dependent enzymes is a well known phenomenon in carcinogenesis [52]. An estimation of lipid peroxidation products and antioxidants enzymes has accepted them as significant biomarkers of cancer chemoprevention [35]. As shown in Figure 9A, the activity of three antioxidant enzymes in liver tissue decreased in the DEN-treated group compared to normal control group. However, the administrations of GEE8080 both at 10 and 100 mg/kg b.w. resulted in significant increases of the antioxidant enzymes activity and similar result were observed in the silymarin treated group (100 mg/kg b.w.). The inductions of SOD and GPx enzyme activity by GEE8080 at 10 mg/kg b.w. were slightly greater than those at 100 mg/kg b.w. In addition to the activity of the hepatic antioxidant enzymes, we evaluated the protein expression levels in the liver tissue after GEE8080 treatment (Figure 9B). The DEN-treated group displayed significant decreases in the expression of SOD, GPx and CAT compared to normal control group. The administration of GEE8080 especially at 10 mg/kg significantly restored the DEN-reduced expression of these enzymes. However, the higher dose of 100 mg/kg failed to restore the DEN-reduced enzymes expression. The reason(s) for the difference at higher dose is not clear, but it could be due to involvement of other mechanisms such as cytotoxic effect at the higher dose, and further side-by-side studies on the dose effect would be needed. Based on these findings, it is likely that the protection by GEE8080, at least at the dose of 10 mg/kg, against DEN-induced hepatotoxicity is associated with the induction of antioxidant enzymes through the induction of Nrf2 pathway. 

## 3. Experimental

### 3.1. Reagents

Fresh ginger rhizomes (*Zingiber officinale*, Roscoe) were acquired from a local farm in Busan, South Korea in March, 2008 and all the samples were stored in a freezer at 80 °C prior to experiments. Antibodies against Nrf2, β-actin and horseradish peroxidase-conjugated anti-rabbit and anti-goat immunoglobulin IgG were purchased from Santa Cruz Biotechnology Inc. (Santa Cruz, CA, USA); HO-1 from Calbiochem (Darmastadt, Germany); and p-ERK, p-JNK, p-Akt, p-p38, LY294002, UO0126, SP600125, and SB202190 from Cell Signaling Technology (Beverly, MA, USA). MTT [3-(4,5-Dimethylthiazol-2-yl)-2,5-Diphenyltetrazolium Bromide], diethylnitrosamine (DEN), silymarin and thiobarbituric acid (TBA) were purchased from Sigma-Aldrich (Saint Louis, MO, USA). The colorimetric aspartate trasaminase (AST) and alanine transaminase (ALT) assay kits were provided by Young-Dong Co. (Seoul, Korea). 6-Shogaol was purchased from Chromadex Inc. (Irvine, CA, USA). All other reagents and solvents used in this study were of the highest quality commercially available. 

### 3.2. Preparation of Ginger Extracts

After washing to remove debris, ginger samples were sliced using a mechanical slicer and spread on trays for drying at 80 °C in a convection drying oven (VISION, Kyungki, Korea). The dried samples were then ground with a mortar and pestle prior to extraction. Ten grams of the ginger were extracted with 95% ethanol (100 mL) for 24 h either at RT (GEE80RT) or 80 °C using a reflux apparatus (GEE8080). The ginger extracts were filtered through Whatman No.1 filter paper and the filtrates were centrifuged in a Supra22K (Hanil, Incheon, Korea) at 5,500 ± 100 rpm for 10 min at 4 °C. The supernatant was subsequently filtered through a 0.2 µm nylon membrane filter and then concentrated by rotatory evaporation (EYELA, Tokyo, Japan) under 40 °C. The 6-shogaol contents of ginger extracts were analyzed by an Agilent HPLC system (Foster, CA, USA) consisting of a quaternary pump, Photodiode Array Detector, and autosampler. Chromatographic analysis was performed on a 250 × 4.6 mm, 5 μm Alltima HP C18 reversed phase column from Grace (Deerfield, IL, USA) with acetonitrile-water (v/v) as the mobile phase. The HPLC operating parameters were as flows: Injection volume, 5 μL; column flow rate, 1.0 mL/min; chromatographic run time, 48 min; UV spectra recording, 230 nm. The mobile phase was consisted of (A) water and (B) acetonitrile. The gradient elution was as follows; 0 min 45% B, 8 min 50% B, 17 min 65% B, 32 min 100% B, 38 min 100% B, 43 min 45% B, 48 min 45% B. The quantification was carried out using the external standard method. The contents of 6-shogaol from gingers were determined using a calibration graphic established with dilutions of each standard at concentrations ranging from 0.1 mM to 1 mM injected into the HPLC system (correlation coefficient ≥ 0.996).

### 3.3. Cell Culture

The HepG2 human hepatoma cell line was purchased from American Type Culture Collection (ATCC, Manassas, VA, USA) were cultured in F-12 medium supplemented with 10% FBS, 100 units/mL penicillin, 100 µg/mL streptomycin, 1% essential amino acids and 0.1% insulin in a humidified atmosphere containing 5% CO2 in air at 37 °C. HepG2 cells were cultured for 24 h (about 80 to 90% confluency) and the cells were starved overnight with serum-free F-12 media prior to treatments with either vehicle (DMSO, 0.1%) or GEE80RT and GEE8080. 

### 3.4. Animals and Experimental Design

Male Balb/c mice (6 weeks-old) were obtained from Hyochang Science (Daegu, Korea). Animals were provided standard pellet diet and distilled water *ad libitum* and maintained on a 12 h light/dark cycle. The protocol for all animal experiments was approved by the Animal Care and Used Committee of Inje University. The animals were acclimatized for seven days before experiments were performed. The mice were divided into five groups (n = 5 per group). The normal control group received only polyethylene glycol oil (PEG) as vehicle for 1 weeks; the DEN group as a negative control received PEG for one week and then injected *i.p.* with DEN dissolved in saline (30 mg/kg b.w.) three times per week for three weeks [53]; GEE8080 groups were administered with 10 or 100 mg/kg of GEE8080, *p.o.* three times per week for one week and then injected *i.p.* with DEN three times per week for three weeks; silymarin group served as a positive control and was administered with 100 mg/kg of silymarin, *p.o.* three times per week for one week and then injected *i.p.* with DEN three times per week for three weeks.

### 3.5. Cell Viability Assay in Cells

Cell viability was determined using the MTT assay. In the experiments, HepG2 cells were cultured 1 × 10^5^ cells in each well of 24-well plates. MTT is a tetrazolium salt that is cleaved to formazan by the mitochondrial respiratory chain enzyme succinate dehydrogenase, which is active in the live cells. Cells were allowed to grow for 24 h (about 80 to 90% confluency) in complete media. Then, the cells were starved for overnight with serum-free F-12 media. After starvation, media were removed and replaced culture media containing 50 µL of 5 mg/mL MTT. After incubation for 4 h at 37 in a humidified 5% CO_2_ atmosphere, the absorbance was measured at 570 nm with an ELISA reader from BioTek (Winooski, VT, USA). 

### 3.6. ARE-reporter Gene Activity Assay in Cells

HepG2-C8 cell line donated by Dr. Ah-Ng Tony Kong (Rutgers University, Piscataway, NJ, USA) is a stable transfectant of HepG2 cells with pARE-T1-luciferase reporter gene. The ARE-luciferase activity was determined using a luciferase reporter assay system (Promega, Madison, WI, USA). Briefly, after treatments, the cells were washed twice with ice-cold phosphate buffered-saline (pH 7.4) and harvested in 1× reporter lysis buffer. After centrifugation at 13,000 rpm for 10 min, a 20 µL aliquot of the supernatant were mixed with 100 µL of luciferase assay substrate and measured for luciferase activity by using a GloMax luminometer (Promega). The luciferase activity was normalized against protein amount, determined by BCA protein assay from Pierce (Rockford, IL, USA), and expressed as fold of induction over the luciferase activity of control vehicle-treated cells.

### 3.7. Western Blot Analysis

In cells, treated HepG2 cells with GEE80RT, GEE8080 and SFN were washed with 1X-ice cold PBS (pH 7.4). The cells lysed in 1X whole cell and harvested using a cell scraper. In mice, liver tissue of mice was homogenized in cell lysis buffer. The collected cells and homogenated liver tissues were centrifuged at 13,000 rpm and the pellets collected. The protein concentration of each sample was determined using a BCA protein assay kit (Pierce). Proteins were electrophoresed on SDS-PAGE gels, and transferred to polyvinylidene fluoride membranes with a semidry transfer system from Bio-Rad (Hercules, CA, USA). The membrane was blocked in 5% non-fat milk solution for 3 h at RT, and then incubated overnight at 4 °C with indicated primary antibodies. After hybridization with primary antibody, the membrane was incubated with horseradish peroxidase conjugated secondary antibody for 1 h at RT. Final detection was performed with enhanced chemiluminescence Western blotting reagents from Santa Cruz. 

### 3.8. Measurement of AST and ALT Activities in Serum of Mice

Blood samples were centrifuged at 3,000 rpm for 10 min at 4 °C to obtain serum [54]. Serum levels of AST and ALT were determined using commercially available kits (Young-Dong Co., Seoul, Korea).

### 3.9. Measurement of Lipid Peroxidation

Lipid peroxidation was measured by the formation of thiobarbituric acid reactive substances (TBARS) [55]. The liver homogenates were mixed with 4.5% trichloroacetic acid, 0.45% thiobarbituric acid, 7.5% acetic acid and heated for 30 min at 100 °C. After cooling, the precipitate was removed by centrifugation. The absorbance of the sample was measured at 532 nm using a microplate reader (BioTek). Malondialdehyde, obtained by acid hydrolysis of 1,1,3,3-tetraethoxypropane, was used as the standard for the quantification of TBARS. Data was expressed as nmol of MDA.

### 3.10. Assessment of SOD-like Activity in Liver Tissues

Liver homogenates were prepared in cold cell lysis buffer using a homogenizer. The unbroken cells and cell debris were removed by centrifugation at 13,000 rpm for 10 min at 4 °C. The protein concentration for liver tissues was determined using the BCA protein assay (Pierce Biotechnology, Inc., Rockford, IL, USA) according to the manufacturer’s instructions. The supernatant was used immediately for the assays of SOD, GPx and CAT. SOD-like activity was determined by the method SOD-like developed by Liu *et al.* [56]. The reaction mixture was buffered with 50 mM sodium carbonate buffer (pH 10.2) containing 3 mM xanthine, 0.75 mM nitro blue tetrazolium, 3 mM EDTA and 50 µL cell lysates. After pre-incubation for 30 min at RT, the reaction was initiated by adding 50 µL xanthine oxidase (0.1 mg/mL) and stopped by addition of 6 mM copper chloride, and centrifuged at 1,500 g for 15 min. The absorbance of the reaction mixture was measured at 560 nm. 

### 3.11. Assessment of GPx and CAT Activity in Liver Tissues

GPx activity was evaluated by the method of Boddanska *et al. *[57]. The reaction mixture consisted of 0.1 M phosphate buffer (pH 7.0), 1 mM EDTA, 10 mM glutathione, 2 mM sodium azide (NaN3), 1 unit of glutathione reductase, 1.5 mM NADPH and cell lysates. The activity was calculated using the molar extinction coefficient for NADPH of 6.22 µmol/cm at 340 nm. CAT activity was measured according to the method of Carriollo *et al. *[58]. The supernatant (0.1 mL) was added to cuvette containing 1.9 mL of 50 mM phosphate buffer (pH 7.0). Reaction was started by addition of 1.0 mL of freshly prepared 30 mM hydrogen peroxide (H_2_O_2)_. The rate of decomposition of H_2_O_2_ was measured at 240 nm for 1 min. The enzyme activity was expressed as U/mg protein. 

### 3.12. Graphs and Statistical Analysis

The data were expressed at the means ± S.D. The statistical analysis was performed using the SigmaPlot 10.0 program (Systat Software Inc., Chicago, IL, USA). Values were compared to control using analysis of variance (ANOVA) followed by unpaired Student’s *t*-test. *p* < 0.05 were considered as significant.

## 4. Conclusions

The present study demonstrates for the first time the antioxidant defense activity of 6-shogaol-rich ginger extract via the induction of Nrf2/ARE-mediated antioxidant enzyme HO-1 coupled with p38 MAPK pathway in cell line level. Our *in vivo* data also prove the hepatoprotective effects of the 6-shogaol-rich ginger extract through its ability to attenuate the DEN-mediated elevation of liver damage markers such as AST, ALT and lipid peroxidation. The ginger extract also upregulated both the activity and protein expression of hepatic antioxidant enzymes including SOD, GPx and CAT. Although we assume that the biological activities of our ginger extract results from the enriched 6-shogaol content, there might be other bioactive compounds produced by heat during the extraction process, which should be further studied. Taken together, the 6-shogaol-rich ginger extract may be used as a potential antioxidant defense agent. 

## References

[1] Bak M.J., Jun M., Jeong W.S. (2012). Antioxidant and hepatoprotective effects of the red ginseng essential oil in H_2_O_2_-treated HepG2 cells and CCl_4_-treated mice. Int. J. Mol. Sci..

[2] Jeong W.S., Jun M., Kong A.N. (2006). Nrf2: A potential molecular target for cancer chemoprevention by natural compounds. Antioxid. Redox Sign..

[3] Rushmore T.H., Kong A.N. (2002). Pharmacogenomics, regulation and signaling pathways of phase I and II drug metabolizing enzymes. Curr. Drug Metab..

[4] Itoh K., Chiba T., Takahashi S., Ishii T., Igarashi K., Katoh Y., Oyake T., Hayashi N., Satoh K., Hatayama I. (1997). An Nrf2/small Mafheterodimer mediates the induction of phase II detoxifying enzyme genes through antioxidant response elements. Biochem. Biophys. Res. Commun..

[5] Kong A.N., Yu R., Hebbar V., Chen C., Owuor E., Hu R., Ee R., Mandlekar S. (2001). Signal transduction events elicited by cancer prevention compounds. Mutat. Res..

[6] Zipper L.M., Mulcahy R.T. (2000). Inhibition of ERK and p38 MAP kinases inhibits binding of Nrf2 and induction of GCS genes. Biochem. Biophys. Res. Commun..

[7] Afzal M., Al-Hadidi D., Menon M., Pesek J., Dhami M.S. (2001). Ginger: An ethnomedical, chemical and pharmacological review. Drug Metabol. Drug Interact..

[8] Thomson M., Al-Qattan K.K., Al-Sawan S.M., Alnaqeeb M.A., Khan I., Ali M. (2002). The use of ginger (*Zingiber officinale *Rosc.) as a potential anti-inflammatory and antithrombotic agent. Prostaglandins Leukot. Essent. Fatty Acids.

[9] Shukla Y., Singh M. (2007). Cancer preventive properties of ginger: A brief review. Food Chem. Toxicol..

[10] Chen C.Y., Cheng K.C., Chang A.Y., Lin Y.T., Hseu Y.C., Wang H.M. (2012). 10-shogaol, an antioxidant from zingiberofficinale for skin cell proliferation and migration enhancer. Int. J. Mol. Sci..

[11] Suekawa M., Ishige A., Yuasa K., Sudo K., Aburada M., Hosoya E. (1984). Pharmacological studies on ginger. I. Pharmacological actions of pungent constitutents, (6)-gingerol and (6)-shogaol. J. Pharmacobiodyn..

[12] Koo K.L., Ammit A.J., Tran V.H., Duke C.C., Roufogalis B.D. (2001). Gingerols and related analogues inhibit arachidonic acid-induced human platelet serotonin release and aggregation. Thromb. Res..

[13] Ippoushi K., Azuma K., Ito H., Horie H., Higashio H. (2003). [6]-Gingerol inhibits nitric oxide synthesis in activated J774.1 mouse macrophages and prevents peroxynitrite-induced oxidation and nitration reactions. Life Sci..

[14] Kundu J., Surh Y.-J. (2009). Molecular basis of chemoprevention with dietary phytochemicals: Redox-regulated transcription factors as relevant targets. Phytochem. Rev..

[15] Kim E.C., Min J.K., Kim T.Y., Lee S.J., Yang H.O., Han S., Kim Y.M., Kwon Y.G. (2005). [6]-Gingerol, a pungent ingredient of ginger, inhibits angiogenesis *in vitro* and *in vivo*. Biochem. Biophys. Res. Commun..

[16] Weng C.J., Wu C.F., Huang H.W., Ho C.T., Yen G.C. (2010). Anti-invasion effects of 6-shogaol and 6-gingerol, two active components in ginger, on human hepatocarcinoma cells. Mol. Nutr. Food Res..

[17] Peng F., Tao Q., Wu X., Dou H., Spencer S., Mang C., Xu L., Sun L., Zhao Y., Li H. (2012). Cytotoxic, cytoprotective and antioxidant effects of isolated phenolic compounds from fresh ginger. Fitoterapia.

[18] Wu H., Hsieh M.C., Lo C.Y., Liu C.B., Sang S., Ho C.T., Pan M.H. (2010). 6-Shogaol is more effective than 6-gingerol and curcumin in inhibiting 12-O-tetradecanoylphorbol 13-acetate-induced tumor promotion in mice. Mol. Nutr. Food Res..

[19] Dugasani S., Pichika M.R., Nadarajah V.D., Balijepalli M.K., Tandra S., Korlakunta J.N. (2010). Comparative antioxidant and anti-inflammatory effects of [6]-gingerol, [8]-gingerol, [10]-gingerol and [6]-shogaol. J. Ethnopharmacol..

[20] Bhattarai S., Tran V.H., Duke C.C. (2001). The stability of gingerol and shogaol in aqueous solutions. J. Pharm. Sci..

[21] Zhang Y., Kensler T.W., Cho C.G., Posner G.H., Talalay P. (1994). Anticarcinogenic activities of sulforaphane and structurally related synthetic norbornylisothiocyanates. Proc. Natl. Acad. Sci. USA.

[22] Surh Y.J. (2003). Cancer chemoprevention with dietary phytochemicals. Nat. Rev. Cancer.

[23] Ryter S.W., Alam J., Choi A.M. (2006). Heme oxygenase-1/carbon monoxide: From basic science to therapeutic applications. Physiol. Rev..

[24] Loboda A., Jazwa A., Grochot-Przeczek A., Rutkowski A.J., Cisowski J., Agarwal A., Jozkowicz A., Dulak J. (2008). Heme oxygenase-1 and the vascular bed: From molecular mechanisms to therapeutic opportunities. Antioxid. Redox Signal..

[25] Hsu C.L., Wu Y.L., Tang G.J., Lee T.S., Kou Y.R. (2009). Ginkgo biloba extract confers protection from cigarette smoke extract-induced apoptosis in human lung endothelial cells: Role of heme oxygenase-1. Pulm. Pharmacol. Ther..

[26] Hu R., Xu C., Shen G., Jain M.R., Khor T.O., Gopalkrishnan A., Lin W., Reddy B., Chan J.Y., Kong A.N. (2006). Identification of Nrf2-regulated genes induced by chemopreventiveisothiocyanate PEITC by oligonucleotide microarray. Life Sci..

[27] Kweon S., Park K.A., Choi H. (2003). Chemopreventive effect of garlic powder diet in diethylnitrosamine-induced rat hepatocarcinogenesis. Life Sci..

[28] Keum Y.S., Yu S., Chang P.P., Yuan X., Kim J.H., Xu C., Han J., Agarwal A., Kong A.N. (2006). Mechanism of action of sulforaphane: Inhibition of p38 mitogen-activated protein kinaseisoforms contributing to the induction of antioxidant response element-mediated heme oxygenase-1 in human hepatoma HepG2 cells. Cancer Res..

[29] McNally S.J., Harrison E.M., Ross J.A., Garden O.J., Wigmore S.J. (2007). Curcumin induces hemeoxygenase 1 through generation of reactive oxygen species, p38 activation and phosphatase inhibition. Int. J. Mol. Med..

[30] Gong P., Hu B., Cederbaum A.I. (2004). Diallyl sulfide induces heme oxygenase-1 through MAPK pathway. Arch. Biochem. Biophys..

[31] Jeong W.S., Keum Y.S., Chen C., Jain M.R., Shen G., Kim J.H., Li W., Kong A.N. (2005). Differential expression and stability of endogenous nuclear factor E2-related factor 2 (Nrf2) by natural chemopreventive compounds in HepG2 human hepatoma cells. J. Biochem. Mol. Biol..

[32] Bak M.J., Jun M., Jeong W.S. (2012). Procyanidins from wild grape (Vitisamurensis) seeds regulate ARE-mediated enzyme expression via Nrf2 coupled with p38 and PI3K/Akt pathway in HepG2 cells. Int. J. Mol. Sci..

[33] Yu R., Lei W., Mandlekar S., Weber M.J., Der C.J., Wu J., Kong A.N. (1999). Role of a mitogen-activated protein kinase pathway in the induction of phase II detoxifying enzymes by chemicals. J. Biol. Chem..

[34] Shen G., Hebbar V., Nair S., Xu C., Li W., Lin W., Keum Y.S., Han J., Gallo M.A., Kong A.N. (2004). Regulation of Nrf2 transactivation domain activity. The differential effects of mitogen-activated protein kinase cascades and synergistic stimulatory effect of Raf and CREB-binding protein. J. Biol. Chem..

[35] Hayes J.D., Pulford D.J. (1995). The glutathione S-transferase supergene family: Regulation of GST and the contribution of the isoenzymes to cancer chemoprotection and drug resistance. Crit. Rev. Biochem. Mol. Biol..

[36] Hwang Y.P., Choi J.H., Choi J.M., Chung Y.C., Jeong H.G. (2011). Protective mechanisms of anthocyanins from purple sweet potato against tert-butyl hydroperoxide-induced hepatotoxicity. Food Chem. Toxicol..

[37] Hwang Y.P., Jeong H.G. (2008). Mechanism of phytoestrogenpuerarin-mediated cytoprotection followingoxidative injury: Estrogen receptor-dependent up-regulation of PI3K/Akt and HO-1. Toxicol. Appl. Pharmacol..

[38] Kang J.S., Wanibuchi H., Morimura K., Gonzalez F.J., Fukushima S. (2007). Role of CYP2E1 in diethylnitrosamine-induced hepatocarcinogenesis *in vivo*. Cancer Res..

[39] Chuang S.E., Cheng A.L., Lin J.K., Kuo M.L. (2000). Inhibition by curcumin of diethylnitrosamine-induced hepatic hyperplasia, inflammation, cellular gene products and cell-cycle-related proteins in rats. Food Chem. Toxicol..

[40] Kohle C., Schwarz M., Bock K.W. (2008). Promotion of hepatocarcinogenesis in humans and animal models. Arch. Toxicol..

[41] Naik S.R., Panda V.S. (2007). Antioxidant and hepatoprotective effects of Ginkgo bilobaphytosomes in carbon tetrachloride-induced liver injury in rodents. Liver Int..

[42] Kalantari H., Salehi M. (2001). The protective effect of garlic oil on hepatotoxicity induced by acetaminophen in mice and comparison with N-acetylcysteine. Saudi. Med. J..

[43] Benzie I.F. (1996). Lipid peroxidation: A review of causes, consequences, measurement and dietary influences. Int. J. Food Sci. Nutr..

[44] Singh B.N., Singh B.R., Sarma B.K., Singh H.B. (2009). Potential chemoprevention of N-nitrosodiethylamine-induced hepatocarcinogenesis by polyphenolics from Acacia nilotica bark. Chem. Biol. Interact..

[45] Dianzani M.U. (1989). Lipid peroxidation and cancer: A critical reconsideration. Tumori.

[46] Shaarawy S.M., Tohamy A.A., Elgendy S.M., Elmageed Z.Y., Bahnasy A., Mohamed M.S., Kandil E., Matrougui K. (2009). Protective effects of garlic and silymarin on NDEA-induced rats hepatotoxicity. Int. J. Biol. Sci..

[47] Nguyen T., Nioi P., Pickett C.B. (2009). The Nrf2-antioxidant response element signaling pathway and its activation by oxidative stress. J. Biol. Chem..

[48] Perrella M.A., Yet S.F. (2003). Role of heme oxygenase-1 in cardiovascular function. Curr. Pharm. Des..

[49] Prawan A., Kundu J.K., Surh Y.J. (2005). Molecular basis of heme oxygenase-1 induction: Implications for chemoprevention and chemoprotection. Antioxid. Redox. Sign..

[50] Sabina E.P., Pragasam S.J., Kumar S., Rasool M. (2011). 6-gingerol, an active ingredient of ginger, protects acetaminophen-induced hepatotoxicity in mice. ZhongXi Yi Jie He Xue Bao.

[51] Banakar M.C., Paramasivan S.K., Chattopadhyay M.B., Datta S., Chakraborty P., Chatterjee M., Kannan K., Thygarajan E. (2004). 1α,25-dihydroxyvitamin D3 prevents DNA damage and restores antioxidant enzymes in rat hepatocarcinogenesis induced by diethylnitrosamine and promoted by phenobarbital. World J. Gastroenterol..

[52] Anis K.V., Rajeshkumar N.V., Kuttan R. (2001). Inhibition of chemical carcinogenesis by berberine in rats and mice. J. Pharm. Pharmacol..

[53] Gurski R.R., Schirmer C.C., Kruel C.R., Komlos F., Kruel C.D., Edelweiss M.I. (1999). Induction of esophageal carcinogenesis by diethylnitrosamine and assessment of the promoting effect of ethanol and N-nitrosonornicotine: Experimental model in mice. Dis. Esophagus.

[54] Jin Y.S., Sa J.H., Shim T.H., Rhee H.I., Wang M.H. (2005). Hepatoprotective and antioxidant effects of *Morus bombycis* Koidzumi on CCl_4_-induced liver damage. Biochem. Biophys. Res. Commun..

[55] Ohkawa H., Ohishi N., Yagi K. (1979). Assay for lipid peroxides in animal tissues by thiobarbituric acid reaction. Anal. Biochem..

[56] Liu F., Ooi V.E., Chang S.T. (1997). Free radical scavenging activities of mushroom polysaccharide extracts. Life Sci..

[57] Bogdanska J.J., Korneti P., Todorova B. (2003). Erythrocyte superoxide dismutase, glutathione peroxidase and catalase activities in healthy male subjects in Republic of Macedonia. Bratisl. Lek. Listy.

[58] Carrillo M.C., Kanai S., Nokubo M., Kitani K. (1991). (-)Deprenyl induces activities of both superoxide dismutase and catalase but not of glutathione peroxidase in the striatum of young male rats. Life Sci..

